# VR-Aided Ankle Rehabilitation Decision-Making Based on Convolutional Gated Recurrent Neural Network

**DOI:** 10.3390/s24216998

**Published:** 2024-10-30

**Authors:** Hu Zhang, Yujia Liao, Chang Zhu, Wei Meng, Quan Liu, Sheng Q. Xie

**Affiliations:** 1 School of Information Engineering, Wuhan University of Technology, Wuhan 430070, China; huzhang@whut.edu.cn (H.Z.); lyj15972557539@163.com (Y.L.); changzhu@whut.edu.cn (C.Z.); quanliu@whut.edu.cn (Q.L.); 2 School of Electronic and Electrical Engineering, University of Leeds, Leeds LS2 9JT, UK

**Keywords:** stroke, rehabilitation, rehabilitation decision-making, convolutional gated recurrent neural network, whale optimization algorithm

## Abstract

Traditional rehabilitation training for stroke patients with ankle joint issues typically relies on the expertise of physicians. However, when confronted with complex challenges, such as online decision-making or assessing rehabilitation progress, even seasoned experts may not anticipate all potential hurdles. A novel approach is necessary—one that effectively addresses these complexities without solely leaning on expert experience. Previous studies have introduced a rehabilitation assessment method based on fuzzy neural networks. This paper proposes a novel approach, which is a VR-aided ankle rehabilitation decision-making model based on a convolutional gated recurrent neural network. This model takes various inputs, including ankle dorsiflexion range of motion, angular velocity, jerk, and motion performance scores, gathered from wearable motion inertial sensors during virtual reality rehabilitation. To overcome the challenge of limited data, data augmentation techniques are employed. This allows for the simulation of five stages of rehabilitation based on the Brunnstrom staging scale, providing tailored control parameters for virtual training scenarios suited to patients at different stages of recovery. Experiments comparing the classification performance of convolutional neural networks and long short-term memory networks were conducted. The results were compelling: the optimized convolutional gated recurrent neural network outperformed both alternatives, boasting an average accuracy of 99.16% and a Macro-F1 score of 0.9786. Importantly, it demonstrated a strong correlation (correlation coefficient r > 0.9) with the assessments made by clinical rehabilitation experts, showing its effectiveness in real-world applications.

## 1. Introduction

According to data from the China National Stroke Survey, the prevalence of stroke among the aged is 2.06%. Stroke often leads to abnormal ankle joint function, including muscle weakness, spasms, and impaired motor control. Therefore, improving motor function is pivotal in post-stroke rehabilitation plans [1]. The restoration of ankle joint function not only facilitates the recovery of walking ability but also mitigates the risk of falls. Rehabilitation robots promote the recovery of damaged muscles and nerves by simulating real physiological movements, assisting patients in rebuilding motor function and muscle control [2,3].

Virtual reality (VR) technology can create immersive and lifelike virtual environments, which include immersion, imagination, and interaction [4]. VR can be categorized into non-immersive and immersive types. Non-immersive VR allows users to interact with the virtual environment, which offers cost-effectiveness, simplicity, and ease of adaptation for rehabilitation training [5]. Immersive VR employs large-screen projections or head-mounted displays to provide users with a fully immersive experience [6,7]. The integration of virtual reality with rehabilitation robots has shown significant efficacy in enhancing motor function among stroke patients. Meta-analyses were conducted by Li et al. [8], comprising 31 randomized controlled studies, which revealed that virtual reality training outperforms conventional rehabilitation training in terms of motor function recovery for stroke patients. Jonna et al. [9] developed a six-degree-of-freedom rehabilitation robot system that integrated virtual reality technology and incorporated engaging virtual games to enhance the enjoyment of rehabilitation training and assist patients in performing complex shoulder and elbow joint movements. Lin et al. [10] devised a guidance control rule with a variable stiffness term, enabling lower limb rehabilitation robots to automatically adjust admittance parameters based on trigger feedback to respond to various virtual events. Most current studies do not automatically output the control parameters of virtual reality corresponding to different rehabilitation stages based on rehabilitation assessment results during rehabilitation training. In addition, the search speed and optimization effect of the model parameters of the adaptive rehabilitation training decision-making model need to be strengthened [11,12].

This paper adopts the WOA-CNN-GRU model for rehabilitation decision-making model, which has efficient feature extraction = and time series modeling abilities. The SMOTE algorithm is adopted to solve the problem of a small or unbalanced dataset in the training process. The whale optimization algorithm is employed to adjust the hyperparameters of the network to improve the search speed of optimal model parameters and performance of the rehabilitation decision-making model. The WOA-CNN-GRU model combines the advantages of various algorithms and can achieve better performance in complex application scenarios.

The structure of this paper is outlined as follows: The second section introduces the ankle rehabilitation system and the clinical rehabilitation training mechanism. The third section encompasses the results obtained from experiments and the evaluation analysis of network performance. The fourth section summarizes the key findings and delves into future research directions. The fifth section offers a concise overview of the paper.

## 2. Methods

### 2.1. Overview of the Proposed System

**Figure 1:**
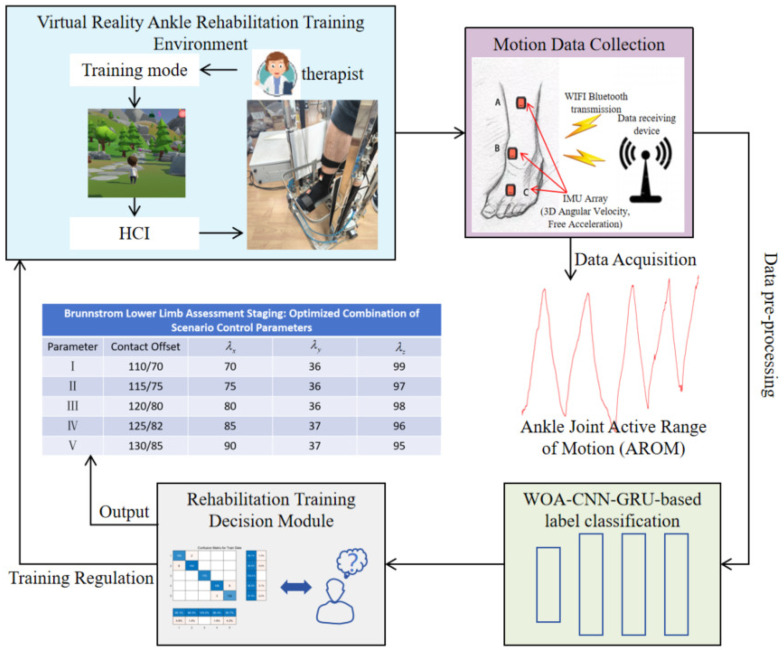
The decision-making algorithm implementation flowchart.

This study introduces a real-time decision-making algorithm for adjusting training intensity within a self-developed virtual reality ankle rehabilitation platform. The algorithm implementation process is depicted in Figure 1. Leveraging convolutional gated recurrent neural networks for machine learning, the algorithm aims to monitor and adapt training intensity across six parameters during rehabilitation sessions. These parameters encompass three ankle kinematic metrics, one fuzzy neural network-based rehabilitation assessment parameter, and two indicators of virtual scene training completion [13].

The algorithm outputs combinations of virtual scene control parameters aligned with stages I–V of the Brunnstrom staging scale, ensuring precise tracking of rehabilitation intensity [14]. The following is a concise overview of the algorithm designed for virtual reality ankle rehabilitation tasks. The system architecture comprises five key modules: (1) the virtual reality ankle rehabilitation platform, (2) the data acquisition module, (3) the rehabilitation training decision module, (4) data preprocessing, and (5) the training regulation module, all dedicated to analyzing patient progress.

In this setup, Xt(z)={xt,st} represents the data sequence of the rehabilitation training cycle at time *t* within virtual rehabilitation scenes labeled z∈{s1,s2,s3}. Here, xt denotes the current training feature parameters, while st indicates the rehabilitation staging category assigned by experts. Initially, the virtual reality ankle rehabilitation system’s microcontroller employs inertial motion sensors and accesses the virtual scene database to capture the training data sequences of patients, extracting six key rehabilitation training parameters. Subsequently, the rehabilitation training decision module categorizes these processed parameters into staging categories. Finally, the repetition counter module regulates virtual scenes using the following approach: considering the current training cycle as the focal point, it examines $xt_{-2}$ and $xt_{-1}$ as the preceding training cycle window and $xt_{+1}$ and $xt_{+2}$ as the subsequent window (assuming each training cycle is effective).

### 2.2. Ankle Rehabilitation Robot

**Figure 2:**
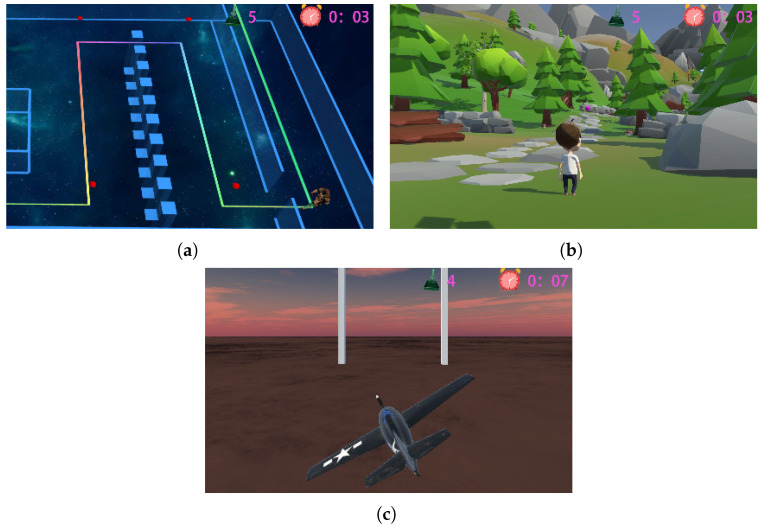
Virtual reality ankle rehabilitation scene. (**a**) Spacewalking. (**b**) Mountain hiking. (**c**) Flight simulation.

Research has shown that patients who are exposed to prolonged periods of uninspiring and repetitive movement patterns may experience subpar rehabilitation outcomes and a lack of motivation to engage in continued rehabilitation training [15]. Figure 2 depicts schematic diagrams of the rehabilitation game virtual scenes. Leveraging Unity3D as the development engine for virtual environments, the system delivers three highly immersive virtual scenes and implements physics-based collision detection tailored for patients with diverse needs, alongside virtual reality mapping for rehabilitation training.

This study employs a method of decomposing the Euler angles of the ankle–foot joint within the real-world coordinate system. This process involves three consecutive rotations around the *x*, *y*, and *z* axes, depicting a single pose transformation of the ankle–foot within the virtual training scene. The representation of the transformation response matrix is as follows:

(1) $$R_{X}\left(\phi \right)=\lambda_{X}\left[\begin{array}{ccc} 1 & 0 & 0\\ 0 & \cos\phi & \sin\phi \\ 0 & -\sin\phi & \cos\phi \end{array}\right]$$

(2) $$R_{Y}\left(\theta \right)=\lambda_{Y}\left[\begin{array}{ccc} \cos\theta & 0 & -\sin\theta \\ 0 & 1 & 0\\ \sin\theta & 0 & \cos\theta \end{array}\right]$$

(3) $$R_{Z}\left(\psi \right)=\lambda_{Z}\left[\begin{array}{ccc} \cos\psi & \sin\psi & 0\\ -\sin\psi & \cos\psi & 0\\ 0 & 0 & 1 \end{array}\right]$$

We obtain the final transformation matrix *A* by stacking these three transformations, reflecting the real-time adjustments:

(4) $$A=R_{X}\left(\phi \right)\times R_{Y}\left(\theta \right)\times R_{Z}\left(\psi \right)$$

**Figure 3:**
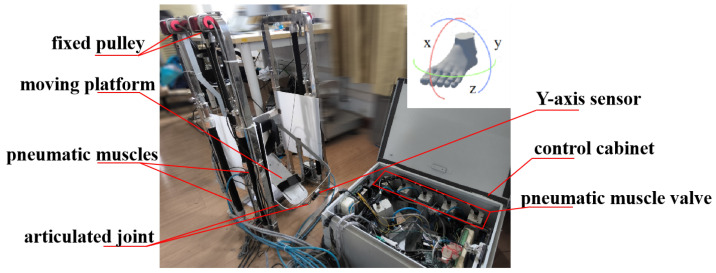
Flexible ankle rehabilitation robot platform.

The core components of the rehabilitation robot consist of pneumatic muscle-driven mechanisms for ankle rehabilitation, a hardware control box, and a PC, as shown in Figure 3. Powered by five FESTO pneumatic muscles, the robot offers three degrees of freedom for basic ankle joint movements. Flexible tendons link the pneumatic muscles to the motion platform, driving it through the contraction motion of the pneumatic muscles. Compared to rigid actuators, pneumatic muscle actuators boast advantages such as lightweight construction, enhanced safety, and superior compliance. Utilizing flexible pneumatic muscle actuators in ankle rehabilitation robots represents a significant advantage [16].

### 2.3. Data Collection

Two healthy adults and four stroke patients volunteered to participate in the experiments, providing written consent prior to their involvement. All subjects gave their informed consent for inclusion before they participated in the study. The study was conducted in accordance with the Declaration of Helsinki, and the protocol was approved by the Ethics Committee of Renmin Hospital of Wuhan University (Project identification code: WDRY2020-K191). Under the guidance of medical experts, all participants engaged in simulated ankle joint dysfunction training, wherein they autonomously maneuvered their ankles through a full range of motions—such as inversion, eversion, dorsiflexion, and plantarflexion—ensuring the stability of the leg while focusing solely on ankle movement. The physicians then provided comprehensive Brunnstrom assessment results to evaluate their performance accurately.

**Figure 4:**
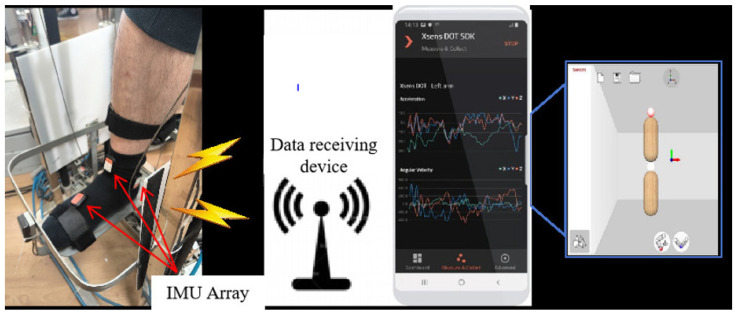
Motion data collection based on Xsens DOT.

**Figure 5:**
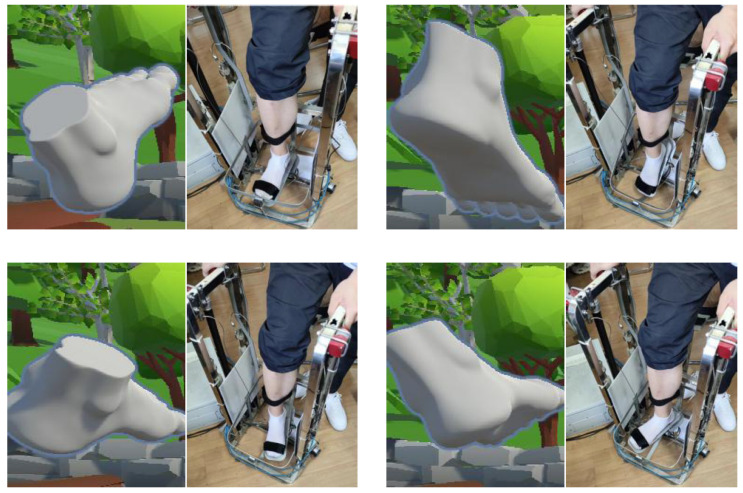
The different actual movements corresponding to virtual reality.

To ensure optimal sensor placement, participants were instructed to wear ankle socks and shorts. Initially, traditional guidance training involved equipping patients with three wireless inertial sensors (IMUs) from Xsens Technologies, Enschede, The Netherlands. These IMUs were strategically positioned on the inner ankle, tarsal center, and thumb joint along the vertical axis, as illustrated in Figure 4. Each IMU unit was securely fastened with a black strap and communicated data via Bluetooth at 60 Hz, capturing precise movement details. Figure 5 shows the ankle–foot dorsiflexion/plantarflexion and inversion/eversion movements mapped in virtual reality.

Subjects were tasked with simulating the lower limb motor dysfunction stages of Brunnstrom I–V as seen in stroke patients. This comprehensive training regimen involved both traditional guidance training and virtual reality rehabilitation. In the traditional approach, patients performed actions such as ankle inversion, eversion, dorsiflexion, and plantarflexion, aiming to achieve key rehabilitation indicators. These actions were executed with the ankle moving at a constant speed of π/6 rad/s until reaching the maximum voluntary range of motion. Patients were required to hold this position for 2 s upon reaching maximum flexion, ensuring minimal muscle compensatory movements by keeping the legs stationary. Each action was conducted 10–15 times for 6 sets with a 30-s rest between sets, totaling a training session of 20–30 min for each lower limb. Motion sensors captured and extracted features such as maximum ankle range of motion (AROM), maximum angular acceleration α, and jerk indicators to derive normalized rehabilitation assessment scores.

Patients, strapped into an ankle rehabilitation robot, navigated virtual environments by controlling a digital avatar. They were instructed follow predefined routes and complete sets of tasks tailored to their initial clinical assessment. Difficulty levels adjusted dynamically; encountering obstacles or exceeding time limits resulted in score deductions. The ankle rehabilitation robot recorded the patient’s performance, and an internal state machine generated a final completion evaluation score. In this study, the patient’s task performance and evaluation data in the virtual scene served as inputs to a decision model. This model predicted the current stage of the rehabilitation cycle based on the Brunnstrom scale. These parameters, including collider settings and virtual reality mapping intensity, automatically adjusted the difficulty of virtual tasks according to the patient’s progress, ensuring engaging and effective rehabilitation.

**Figure 6:**
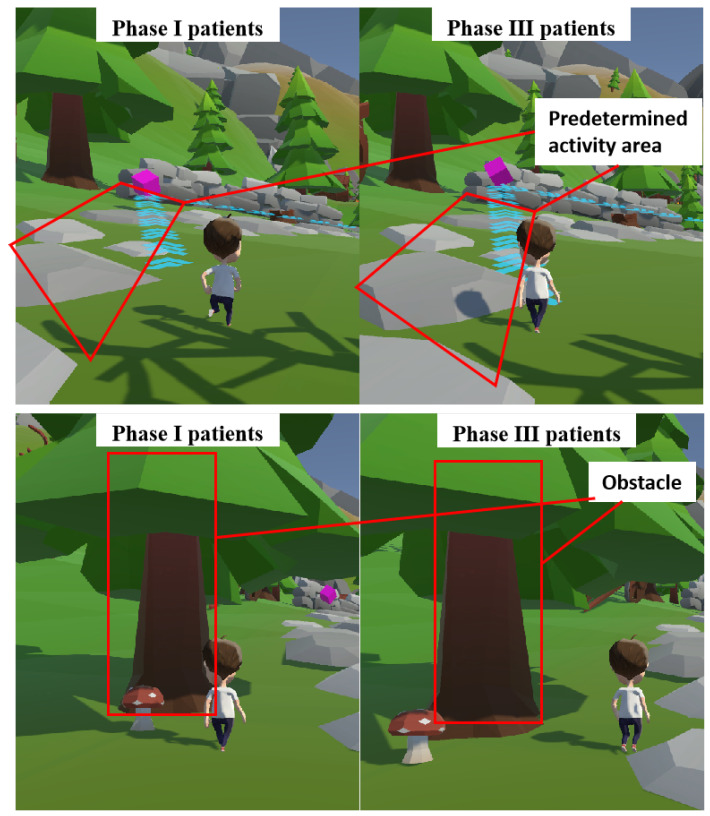
The effectiveness of collision detection for patients at various stages of rehabilitation.

The response coefficients of patients to the three degrees of freedom of the ankle joint ($\lambda_{X}$, $\lambda_{Y}$ and $\lambda_{Z}$) were linked to the sensitivity of robot control. As shown in Figure 6, the system configured different maximum contact distances for obstacles or routes tailored to patients in various stages of rehabilitation.

### 2.4. Rehabilitation Decision-Making Model Based on WOA-CNN-GRU

**Figure 7:**
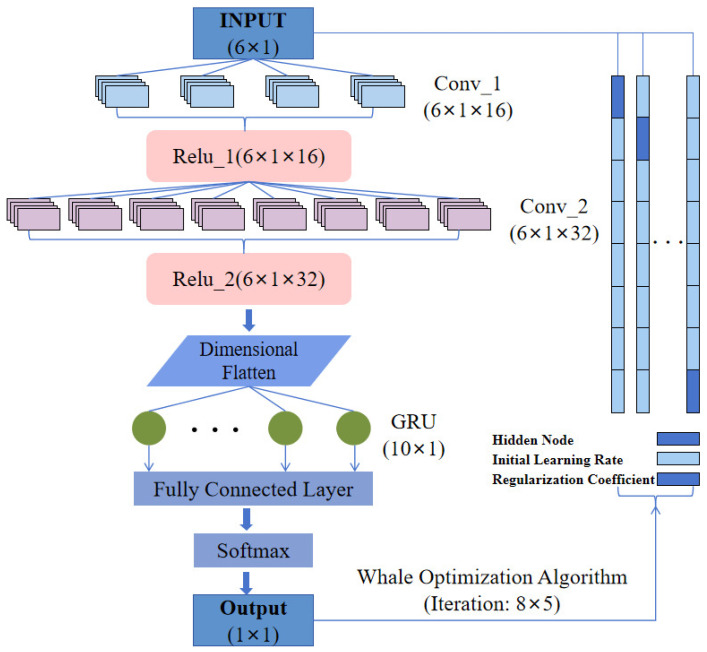
The architecture of the WOA-CNN-GRU network.

The model in this paper utilizes the gated recurrent neural network with gated recurrent units (GRUs) and the convolutional neural network (CNN). Recent studies [17] have demonstrated that GRUs and CNNs exhibit superior learning capabilities when tackling classification tasks in rehabilitation. GRUs tackle issues like vanishing gradients and gradient entanglement, thus bolstering the model’s capacity to handle long-term rehabilitation training sequences and capture dependencies with significant temporal gaps [18]. The architectural framework of the WOA-CNN-GRU network model for rehabilitation decision-making is depicted in Figure 7. Algorithm 1 provides an in-depth explanation of the model training process.
**Algorithm 1** Rehabilitation Decision-making Model based on WOA-CNN-GRU
**Input:** Dataset $D={\left\{EvalScore^{\left(n\right)},T_{c}^{\left(n\right)},Completion^{\left(n\right)}\right\}}_{n=1}^{N}.$ (EvalScore—the normalized
 rehabilitation assessment score of patients, $T_{c}$—the current training completion times collection, Completion—the degree of completion of training tasks)
**Output:** Brunnstrom assessment scale
 Initialization: SearchAgents←8, Max_iteration←5, dim←3, $\mathrm{lb}\leftarrow [1e^{-4},10,1e^{-5}]$,
 $\mathrm{ub}\leftarrow [1e^{-1},30,1e^{-2}]$
 Randomly shuffle dataset *D* and split it into a training set and a test set.
 **repeat**
  **repeat**
   fitness=fobj(SearchAgents_i)   **if** fitness<Best_Cost
**then**   Best_Cost=fitness   Best_pos=SearchAgents_i  **end if**
  **until** i←1<=SearchAgents
  a=2−t∗((2)/Max_iteration)
  **repeat**
   Adjust parameter *a* to modify the speed and direction of the search, thereby
   updating the position of SearchAgent_i.
  **until** i←1<=SearchAgents_i
  t←t+1
  [best_hd,best_lr,best_l2]←Best_pos
  **until** t←1<=Max_iteration  InitialLearnRate←best_lr,L2Regularization←best_l2  **repeat**   running_loss=0.0   self.gru=nn.GRU(input_size=32×1×16, hidden_size=best_hd, num_layers=1)  **repeat**
   outputs=CNN−GRU_net(inputs)
   loss=criterion(outputs,labels)
   $running_{l}oss+=loss.item\left(\right)$  **until** i←1<=Data_Size
 **until** n←1<=Max_Epochs

Through extensive training iterations and optimizations, we meticulously chose the best parameter combination. We assessed the model’s classification performance and its ability to generalize using a variety of evaluation metrics, including accuracy percentage, multi-class performance evaluation index, and the visual representation provided by the confusion matrix graph. Moreover, employing the whale optimization algorithm, we could fine-tune parameters such as the number of hidden layers, initial learning rate, and regularization coefficient [19].

In the initial phase, the prey surrounded by the current whales is regarded as the ideal solution or an approximation of the optimal solution. Assuming a d-dimensional space, the position of the current best whale individual $X^{*}$ is represented as ($X_{1}^{*}$, $X_{2}^{*}$, …, $X_{d}^{*}$) and the position of the whale individual $X^{j}$ is denoted as ($X_{1}^{j}$, $X_{2}^{j}$, …, $X_{d}^{j}$). Then, the formula for calculating the next position $X^{j+1}$ ($X_{1}^{j+1}$, $X_{2}^{j+1}$, …, $X_{d}^{j+1}$) of the whale individual $X^{j}$ under the influence of the best whale individual $X^{*}$ is as follows:

(5) $$X_{k}^{j+1}=X_{k}^{*}-A_{1}\cdot D_{k}$$

(6) $$D_{k}=\left|C_{1}\cdot X_{k}^{*}-X_{k}^{j}\right|$$

(7) $$C_{1}=2r_{2}$$

(8) $$A_{1}=2a\cdot r_{1}-a$$

where $X_{k}^{j+1}$ denotes the *k*-th component of the spatial coordinate $X^{j+1}$. $A_{1}$ stands for the convergence factor coefficient, while $C_{1}$ represents the coefficient vector of the oscillation factor. The parameter *a* gradually decreases from 2 to 0 as the number of iterations increases. Both $r_{1}$ and $r_{2}$ are random numbers ranging from 0 to 1.

In a *d*-dimensional space, suppose the position of the current best whale individual $X^{*}$ is ($X_{1}^{*}$, $X_{2}^{*}$, …, $X_{d}^{*}$) and the position of the whale individual $X^{j}$ is also ($X_{1}^{j}$, $X_{2}^{j}$, …, $X_{d}^{j}$). The current whale individual spirals toward the current best whale individual, a process described by the following equation:

(9) $$X_{k}^{j+1}=X_{k}^{*}+D_{k}\cdot e^{bl}\cdot \cos\left(2\pi l\right)$$

(10) $$D_{k}=\left|X_{k}^{*}-X_{k}^{j}\right|$$

where $D_{k}$ signifies the distance between the current searching individual and the optimal searching individual. Parameter *b* determines the shape of the logarithmic spiral, while *l* is a random number within the interval [−1, 1].

Apart from bubble-net feeding behavior, there is a 50% chance of utilizing encirclement predation. Hence, the mathematical model can be articulated as follows:

(11) $$X_{k}^{j+1}=\left\{\begin{array}{c} X_{k}^{*}-A_{1}\cdot D_{k}\\ X_{k}^{*}+D_{k}\cdot e^{bl}\cdot \cos\left(2\pi l\right) \end{array}\right.\;\;\begin{array}{c} p<0.5\\ p\geq 0.5 \end{array}$$

where *p* is a random probability value ranging from 0 to 1. As the number of iterations, denoted by *j*, increases, the value of the convergence factor $A_{1}$ gradually decreases. Additionally, if |A|<1, each whale progressively converges around the current optimal solution.

In the mathematical model of the predation behavior described above, the convergence factor $A_{1}$ is typically constrained within the range of [−1, 1]. Assuming the current space is d-dimensional, let us denote a random whale individual $X^{rand}$ in the population with position ($X_{1}^{rand}$, $X_{2}^{rand}$, …, $X_{d}^{rand}$), while the position of the whale individual $X^{j}$ is also ($X_{1}^{j}$, $X_{2}^{j}$, …, $X_{d}^{j}$). The mathematical model for search predation behavior is as follows:

(12) $$D_{k}=\left|C_{1}\cdot X_{k}^{rand}-X_{k}^{j}\right|$$

(13) $$X_{k}^{j+1}=X_{k}^{rand}-A_{1}\cdot D_{k}$$

(14) $$C_{1}=2r_{2}$$

(15) $$A_{1}=2a\cdot r_{1}-a$$

where *D* represents the distance between the current whale individual and the randomly selected individual from the population.

Given the limited number of recruited subjects during data collection, this study employs two data augmentation techniques, namely SMOTE and small window segmentation. These methods transform and expand the dataset to generate more training samples.

In the sample collection process, the limited data availability for certain stages of rehabilitation categories may result in an inadequate model understanding of these stages, thus impacting performance [20]. To tackle this challenge, we propose leveraging the SMOTE algorithm to augment the sample size of minority categories and enhance dataset balance. The fundamental steps of this approach are as follows:

a. Begin by selecting samples from the original dataset that belong to the minority class.
b. For each chosen minority class sample, calculate its *k* nearest neighbors using the Euclidean distance metric.
c. Randomly pick one neighbor from the *k* nearest neighbors for each minority class sample and generate a synthetic sample along the line segment connecting them. The synthetic sample *C* is determined using the following formula:
  (16) C=A+λ×(B−A).

In this context, *A* denotes the chosen original sample, while *B* represents a randomly selected sample from its *k* nearest neighbors, with λ being a random number within the range of [0, 1]. For this study, the upsampling rate *k* is set to 100, and the data sampling ratio is adjusted to roughly balance the number of minority class samples with that of the majority class, aiming for a 1:1 ratio.

## 3. Results

In this study, the classification outcomes for six participants are summarized in Table 1. To mitigate incidental biases, each approach underwent five repetitions of training. The findings encompass the test set performance demonstrated by both the proposed method and two advanced benchmark methods, all employing the same data augmentation strategy. The results reveal that CNN-GRU achieved the highest average accuracy across all participants, reaching 99.16%, whereas LSTM displayed the lowest accuracy at 96.08%. Discrepancies in performance among the methods might have originated from their varying adaptability to address specific task complexities. To affirm the superiority of the proposed approach over existing conventional methods, a comparison between the proposed approach and existing methods is conducted, leveraging various algorithmic performance evaluation metrics.

When assessing the performance of classification network models, a number of evaluation metrics are pertinent to this discussion, such as accuracy, precision, and recall. Accuracy (α) indicates the ratio of correctly predicted samples to the total number of samples. Its calculation formula is as follows:

(17) $$accuracy=\frac{\sum_{i=1}^{n}TP_{i}}{\sum_{i=1}^{n}TP_{i}+FP_{i}}$$

where *n* represents the number of classes in the multi-class scenario. $TP_{i}=T_{i}P_{i}$ stands for the number of samples correctly classified as class *i* by the model, while $FP_{i}=\sum_{j=1,j\neq i}^{4}F_{j}P_{i}$ refers to the number of samples incorrectly classified as class *i* when the true class is *j*.

Accuracy serves as a straightforward and intuitive metric for assessing the model’s overall performance. However, accuracy may not provide a fully accurate measure, as the model may exhibit a tendency to favor the more prevalent classes. Hence, a set of evaluation metrics with better balance for addressing class imbalance issues is introduced: macro-Precision, macro-Recall, and macro-F1score.

(18) $$macro-P=\frac{1}{n}\sum^{n}P_{i}$$

(19) $$macro-R=\frac{1}{n}\sum^{n}R_{i}$$

(20) $$macro-F1=\frac{2\times macro-P\times macro-R}{macro-P+macro-R}$$

where $P_{i}$ represents precision rate, calculated as $P_{i}=\frac{TP_{i}}{TP_{i}+FP_{i}}$, and $R_{i}$ stands for recall rate, derived from the equation $R_{i}=\frac{TP_{i}}{TP_{i}+FN_{i}}$.

**Figure 8:**
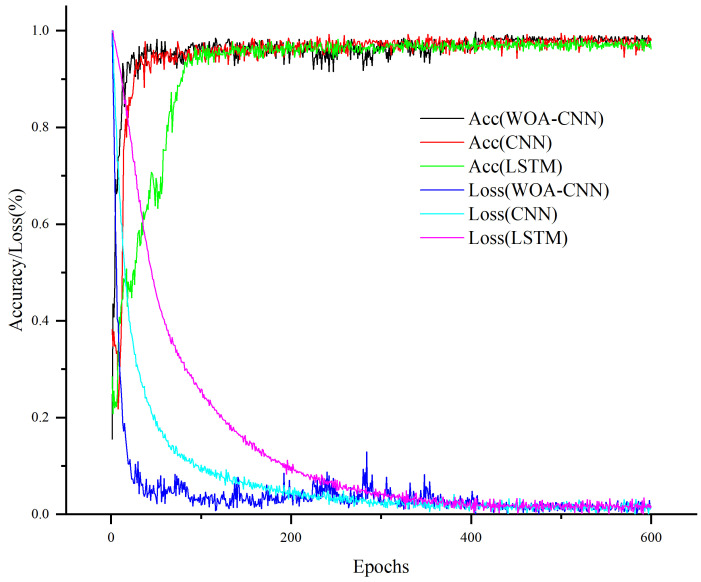
Training loss and accuracy variations of deep learning models across different methods.

**Figure 9:**
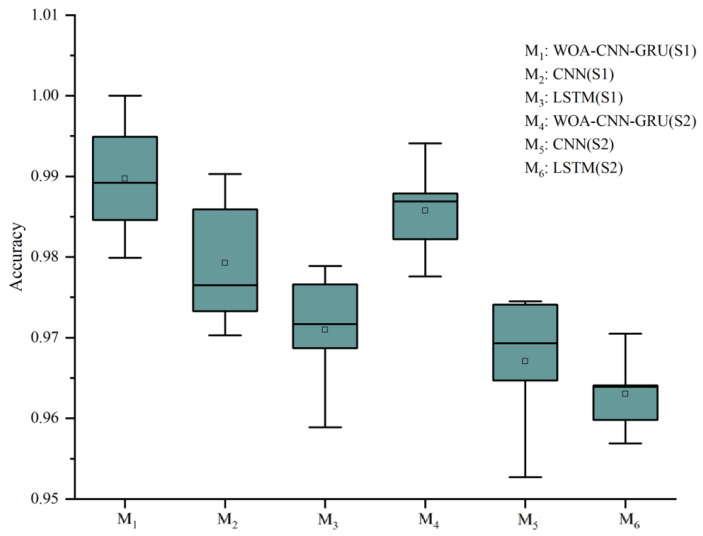
The accuracy of deep learning model.

Table 1 showcases the performance metrics of the three methods with the best hyperparameter combinations, including patient training decision accuracy, macro-Precision, macro-Recall, and macro-F1score. Among these, WOA-CNN-GRU, after evaluating various parameter combinations, exhibited the most optimal performance with a network architecture featuring two convolutional layers, each with a kernel size of 3 × 1. This configuration yielded the highest macro-Precision, macro-Recall, and macro-F1score, indicating its effectiveness in capturing training samples across different stages of ankle joint rehabilitation decision-making and providing comprehensive coverage for each category. Figure 8 illustrates the promising results of the network’s final classification outcomes after 600 epochs of training. In comparison, the two benchmark methods achieved similar accuracies in the range of 97–98%, demonstrating their robust fitting capabilities to the training data. Additionally, Figure 9 illustrates the accuracy results of deep learning models trained multiple times across various methods for multiple subjects, with each square representing the average accuracy of the respective group.

To examine the impact of data augmentation techniques, we expanded the sample data using various configuration combinations and compared the performance of the corresponding augmented samples in the model. The results, shown in Table 2, reveal that the size of the sample data is significantly influenced by the sliding window size and stride. As the stride changes, the sample data size increases almost exponentially. All SMOTE algorithms aim to balance the sample numbers across the six classification categories. However, when the sliding step is set to 100% and the SMOTE data augmentation is 0, indicating the use of original samples without augmentation, the average accuracy is relatively poor. This can be attributed to the model’s inability to effectively capture data features because of the limited input samples. The table clearly illustrates the performance of each category ratio under different augmentation data volumes. Notably, transitioning from a 75% to a 50% sliding window step shows significant performance improvement. Increasing sample diversity enhances the model’s tolerance to outliers, thereby improving its stability. Moreover, when the data augmentation volume is set to 50%, the average accuracy of each method surpasses that of other configurations, suggesting that the model benefits from a relatively small-scale data augmentation.

**Figure 10:**
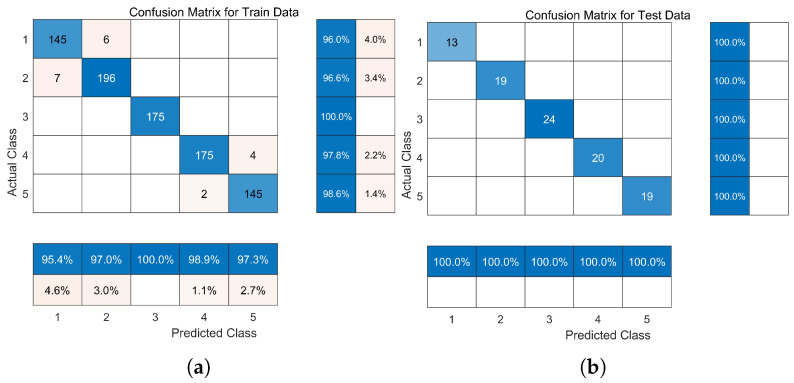

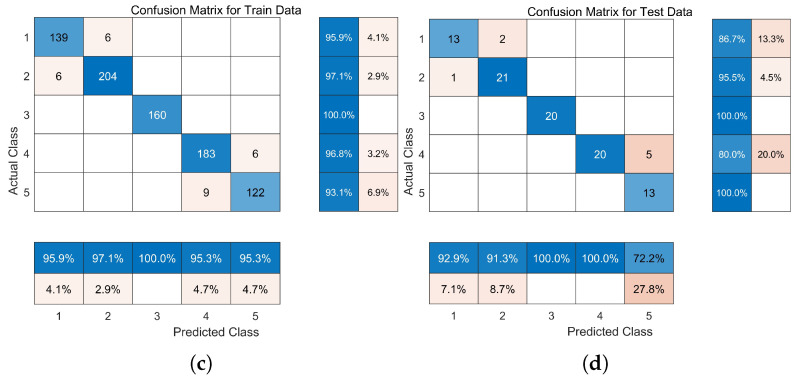
Comparison of optimization algorithms using confusion matrices. (**a**) Training set (WOA). (**b**) Test set (WOA). (**c**) Training set (control group). (**d**) Test set (control group).

The whale optimization algorithm (WOA) was chosen for its efficiency in searching a relatively small search space and its ability to handle optimization objective searches in parallel. Based on the experimental findings mentioned earlier, experiments were conducted using a sliding window step size of 50% and an upsampling rate of 100% as the optimal solution. The WOA optimization group achieved an average classification accuracy of 99.16%, with an average Macro-Precision of 0.9915, average Macro-Recall of 0.9661, and average F1-score of 0.9786. In contrast, the control group’s network hyperparameters were determined based on expert experience, with predefined hidden layer nodes of 10, an initial learning rate of $10^{-3}$, and L2 regularization coefficient of $5\times 10^{-5}$, resulting in an average classification accuracy of 97.55%, average Macro-Precision of 0.9672, average Macro-Recall of 0.8817, and average F1-score of 0.9225. Figure 10 and Table 3 display the classification confusion matrix for the models, indicating the effective enhancement of model prediction and convergence performance through the hyperparameter optimization algorithm.

**Figure 11:**
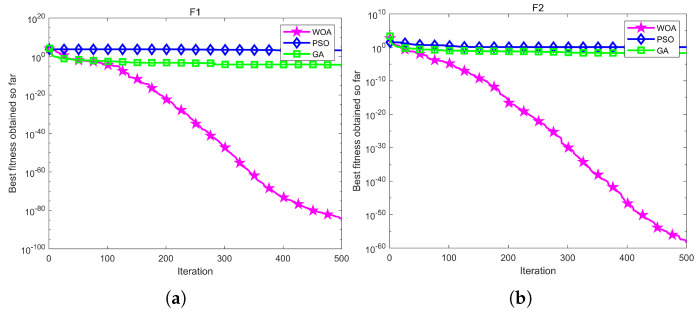

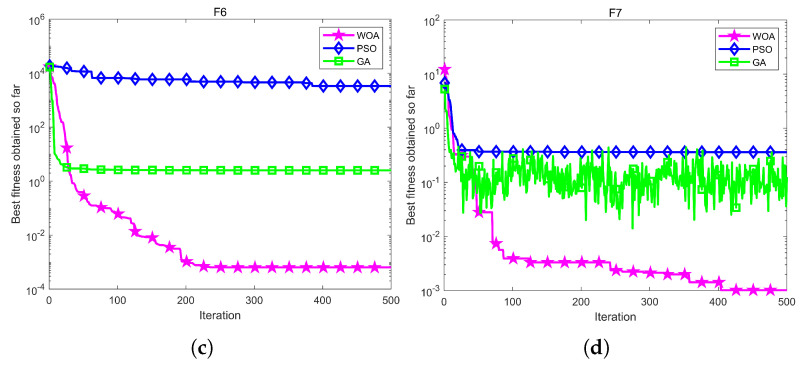
The result of different optimization algorithms based on different test functions. (**a**) F1. (**b**) F2. (**c**) F6. (**d**) F7.

The results of the WOA algorithm are compared with the GA algorithm and PSO algorithm. The PSO algorithm converges quickly, and the algorithm is simple and easy to implement, but it may fall into local optimal solutions. The GA algorithm has a strong global search capability but is computationally intensive and has many iterations. The WOA algorithm is simple and easy to implement and performs well on specific optimization problems. As shown in Figure 11 and Table 4, the WOA algorithm shows excellent searching ability and optimization ability in F1 and F2 one-dimensional test functions, where $F_{1}\left(x\right)=\sum_{i=1}^{n}x_{i}^{2}$ and $F_{2}\left(x\right)=\sum_{i=1}^{n}\left|x_{i}\right|+\prod_{i=1}^{n}\left|x_{i}\right|$, and the WOA algorithm can quickly find the global optimal solution in the two test functions because of the strong evolutionary ability and fast search speed. Furthermore, the WOA algorithm also shows excellent performance in multidimensional test functions, where $F_{6}\left(x\right)=\sum_{i=1}^{n}{\left[x_{i}+0.5\right]}^{2}$ and $F_{7}\left(x\right)=\sum_{i=1}^{n}ix_{i}^{4}+\mathrm{random}\left(0,1\right)$. Multidimensional problems usually have higher complexity and more local optimal solutions, and the WOA algorithm is still able to find the global optimal solution.

## 4. Discussion

This study presents an innovative WOA-CNN-GRU model designed to enhance decision-making in virtual reality ankle rehabilitation training for stroke patients. The model’s innovation lies in its capability to classify features of patient motion data collected during training using non-invasive wearable motion sensor units. Despite having just 400 trainable input samples, the model achieved an impressive classification accuracy of 99.16%, showcasing its remarkable performance in addressing multi-label classification problems, particularly in the realm of decision-making for rehabilitation training programs.

In a virtual reality rehabilitation setting, real-time feedback on patient motion data allows for the dynamic adjustment of training parameters. This means that the difficulty of rehabilitation tasks can be fine-tuned according to this feedback. This highlights the exceptional capability of virtual reality rehabilitation systems to create a closed-loop training environment, enabling seamless interaction between humans and technology. Such interaction not only promotes patient compliance but also ensures ongoing improvements in training effectiveness. Similarly, in real medical practice, healthcare providers pay close attention to these aspects. Rehabilitation specialists must promptly adapt training tasks to address patient discomfort or unexpected events, preventing improper training that can hinder rehabilitation progress or lead to further injuries [23]. Apart from introducing a high-performance rehabilitation training decision-making model, this paper also employed various advanced techniques to provide accurate training feedback. By integrating data augmentation and hyperparameter search algorithms, challenges associated with small or imbalanced datasets during the training process were effectively overcome. Experimental results indicated that adopting smaller sliding window strides could enhance the classification accuracy of the three models, demonstrating improved performance and stability. This confirms the effectiveness of sliding window techniques in capturing local features of time-series data. Additionally, the whale optimization algorithm significantly boosted the classification accuracy and generalization capability of the models in the corresponding experiments. Its efficiency and global search capability enable the models to better adapt to different data features and rehabilitation training scenarios. The integrated application of these two techniques offers valuable insights for addressing multi-label classification problems in rehabilitation training decision-making.

An efficient decision-making model needs to be paired with suitable rehabilitation training environments. This paper designs various virtual training scenarios tailored to patients with different ankle movement characteristics. These scenarios integrate terrain creation tools, physics engine libraries, particle engine libraries, and collision detection components. Through these tools, a virtual rehabilitation training environment for ankle rehabilitation robots was successfully constructed, incorporating appropriate human–machine interaction design. During the initial software debugging phase, the system’s optimization effect was already evident, and further enhancements are planned for the future.

Looking forward, future research will prioritize enhancing the model’s stability and its compatibility with clinical rehabilitation training. Firstly, we will delve deeper into refining parameter configurations. This means fine-tuning and optimizing various parameters within the model to ensure top-notch performance across diverse patient datasets. For instance, we may need to tweak parameters like learning rates, regularization parameters, or network architectures to cater to the unique characteristics and rehabilitation needs of different patient demographics, including age, gender, rehabilitation stage, and goals. Moreover, we will strive to seamlessly integrate these personalized rehabilitation training methods into clinical practice. This will involve close collaboration with clinical physicians and rehabilitation experts to co-create and adjust rehabilitation plans that perfectly fit the actual conditions and requirements of patients. By further boosting the model’s usability and practicality in clinical settings, we aim to deliver even more effective rehabilitation treatments to patients.

## 5. Conclusions

The primary goals of ankle rehabilitation are to boost muscle strength and enhance flexibility, aiming to restore a normal range of motion. However, traditional ankle rehabilitation methods lack real-time monitoring and feedback mechanisms, hindering the timely acquisition of patient training data and rehabilitation progress monitoring. Consequently, therapists struggle to adjust training plans and control training intensity effectively. To address these challenges, this paper proposes a training decision model tailored for ankle rehabilitation robots and their virtual rehabilitation environments. This model leverages performance indicators extracted from patients’ engagement in serious gaming tasks within virtual reality settings to establish an objective classification mapping based on the Brunnstrom staging scale I–V. Ultimately, it outputs corresponding control parameter combinations for rehabilitation scenes. In the experimental phase, we not only analyze classification models employed in other authoritative literature but also explore the impact of model optimization techniques. Overall, the proposed method significantly enhances the personalization and efficiency of ankle rehabilitation training.

## Figures and Tables

**Table 1:** Comparison of WOA-CNN-GRU with benchmark algorithms.

Model	Accuracy	Mac_P	Mac_R	Mac_F1
WOA-CNN-GRU	99.16%	0.9915	0.9661	0.9786
CNN [21]	97.40%	0.9786	0.9162	0.9464
LSTM [22]	96.08%	0.9750	0.9171	0.9452

**Table 2:** Model accuracy varies with different data augmentation configurations.

Window Stride	WOA-CNN-GRU	CNN [21]	LSTM [22]
50 (100%)	0.9752	0.9711	0.9610
35 (70%)	0.9731	0.9794	0.9692
25 (50%)	0.9916	0.9789	0.9895
15 (30%)	0.9910	0.9778	0.9778

**Table 3:** Comparison of WOA algorithm and control group.

Method	Accuracy	Macro-Precision	Macro-Recall	F1-Score
WOA	99.16%	0.9915	0.9661	0.9786
Control group	97.55%	0.9672	0.8817	0.9225

**Table 4:** The result of optimization algorithms based on different test functions.

Function	WOA	GA	PSO
F1	$1.6895\times 10^{-85}$	$1.0052\times 10^{-4}$	2475.6950
F2	$5.7741\times 10^{-58}$	0.0269	1.2004
F6	$7.7469\times 10^{-4}$	2.5192	2301.6360
F7	$7.7469\times 10^{-3}$	0.0529	0.3621

## Data Availability

The data presented in this study are available upon request from the corresponding author. The data are not publicly available due to confidentiality issues.
